# A dual role for the transcription factor Sp8 in postnatal neurogenesis

**DOI:** 10.1038/s41598-018-32134-6

**Published:** 2018-09-28

**Authors:** Elodie Gaborieau, Anahi Hurtado-Chong, Maria Fernández, Kasum Azim, Olivier Raineteau

**Affiliations:** 1 Université Claude Bernard Lyon 1, Inserm, Stem Cell and Brain Research Institute U1208, Bron, France; 2 Brain Research Institute, University of Zürich, Zürich, Switzerland

## Abstract

Neural stem cells (NSCs) of the postnatal subventricular zone (SVZ) continue producing distinct subtypes of olfactory bulb (OB) interneurons throughout life. Understanding the transcriptional coding of this diversity remains a great challenge of modern neurosciences. Interneurons expressing calretinin (CalR) represent the main interneuron subtype produced in the glomerular cell layer (GL) after birth. Previous studies have suggested that their specification relies on expression of the transcription factor Sp8 by SVZ NSCs. In this study, we performed fate mapping of NSCs that generate CalR+ or non-CalR+ interneurons, in order to assess the pattern of Sp8 expression during postnatal neurogenesis. We highlight a complex pattern of Sp8 expression, which appears to be expressed in all interneurons lineages, before getting gradually restricted to maturing CalR+ interneurons. To decipher the early and late functions of Sp8 in postnatal OB neurogenesis, we combined transient, permanent and conditional genetic approaches to manipulate Sp8 at distinct neurogenic stages. While Sp8 plays an early role in controlling proliferation in all lineages, it is not involved in the early specification of CalR+ periglomerular interneurons, but plays a crucial role in their long term survival. Together, our results highlight a crucial and dual role for Sp8 during postnatal neurogenesis.

## Introduction

The ventricular-subventricular zone (V-SVZ) is a brain region of intense germinal activity throughout postnatal life, producing new neurons that migrate and integrate in the olfactory bulb (OB). Its accessibility and regional organization in three microdomains (i.e. lateral, dorsal and medial) that generate distinct interneuron subtypes^1^, makes it an attractive model to study and understand the transcriptional coding of interneuron diversity.

Neuronal specification occurs early in the SVZ, by the expression of specific transcription factors (TFs) in neural stem cells (NSCs) and their immediate progeny. Newly specified neuroblasts then migrate through the rostral migratory stream (RMS) to the OB where they integrate in the glomerular or granular cell layers. Recent studies (reviewed in Deneris and Hobert^2^) have shown that the identity (i.e. neurotransmitter phenotype and connectivity) and the integrity of post-mitotic neurons is actively maintained. Thus, transcription factors, such as those acting in specification, continue to be expressed across the life span of a neuron and act as terminal selector genes.

Calretinin-expressing (CalR+) interneurons are the largest population of OB periglomerular (PG) interneurons produced after birth. They represent 40 to 50% of the whole population of PG interneurons, although their function in olfactory information processing remains to be fully explored^3,4^. Electroporation approaches have revealed a restricted spatial origin of CalR+ PG interneurons^5^. Thus, while CalB+ and TH+ interneurons originate from the lateral and dorsal SVZ respectively, CalR+ interneurons are produced by NSCs from the medial and the dorsal SVZ. Surprisingly, in contrast to CalB+ and TH+ interneurons, limited information exists regarding the TFs that guide CalR+ interneurons specification. Recent studies have revealed a role for Zic TFs in CalR+ PG fate. While Zic3 is expressed in the medial SVZ, Zic1 and Zic2 are expressed in the dorsal SVZ, where they act in inducing CalR+ PG interneurons while repressing a dopaminergic fate^6^. Another TF, the zinc finger TF Sp8 has also been suggested to act in CalR specification^7^. For instance, its conditional deletion in LGE progenitors, which gives the major part of the postnatal SVZ, leads to an impaired OB neurogenesis with a larger impact on CalR+ PG interneurons^7^.

In this study, we combined transcriptional datasets of NSCs isolated from distinct V-SVZ microdomains^8^ and fate mapping of NSCs that generate CalR+ or non-CalR+ interneurons, in order to assess the pattern of Sp8 expression during postnatal neurogenesis. We highlight a complex pattern of Sp8 expression, which appears to be expressed in all interneurons lineages, before getting gradually restricted to maturing CalR+ interneurons in the OB. To decipher the early and late functions of Sp8 in postnatal OB neurogenesis, we combined transient, permanent and conditional genetic approaches to manipulate Sp8 at different neurogenic stages. Our results highlight a dual role for Sp8 during postnatal neurogenesis: i) an early role in proliferation in neuroblasts of all lineages, ii) a role as terminal selector gene that is restricted to mature CalR+ PG interneurons.

## Results

### Sp8 is expressed in neuroblasts of all lineages but gradually become restricted to CalR+ PG interneurons

**Figure 1:**
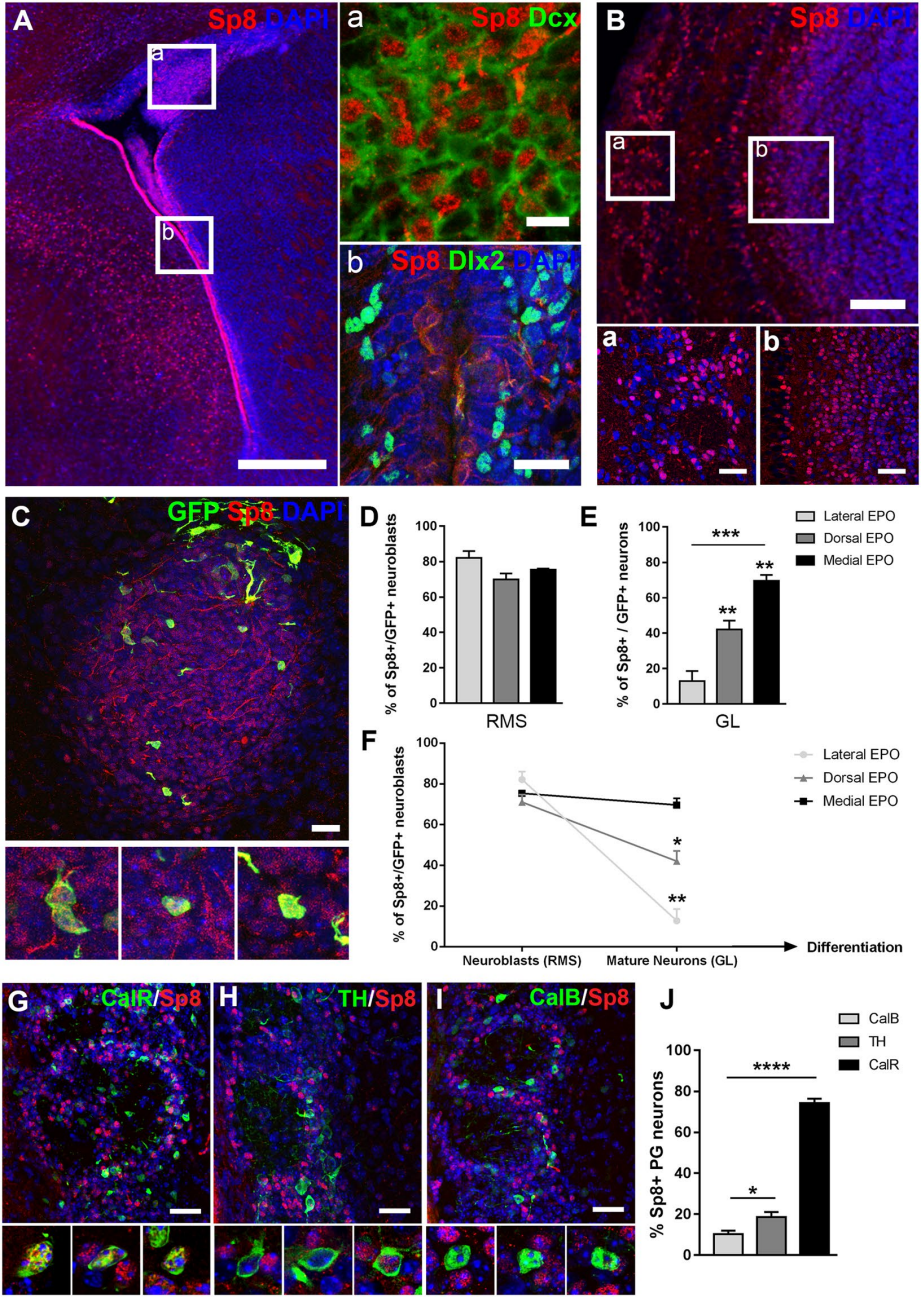
Sp8 expression is observed in neuroblasts originating from all SVZ microdomains but become gradually restricted to mature CalR+ PG interneurons. (**A**) Representative immunostaining revealing Sp8 expression in the postnatal SVZ. The right panel shows nuclear expression in Dcx+ neuroblasts in the RMS (a) while it is absent in Dlx2+ progenitors (b). (**B**) Representative immunostaining of Sp8 in the OB of P23 mice. The two panels below show the specific expression in subpopulations of PG (a) and to a lesser extent granular neurons (b). (**C**–**F**) Electroporation of the 3 microdomains of the SVZ at P2 (i.e. lateral, dorsal and medial) to label neuroblasts of distinct origins and lineages. (**C**) Representative immunostaining of GFP+ labelled migrating neuroblasts in the RMS 4 days post EPO of the medial SVZ. High magnifications show the expression of Sp8 in GFP+ neuroblasts. (**D**–**F**) Quantifications of the proportion of Sp8+/GFP+ migrating neuroblasts in the RMS at 4 dpe (D) and neurons in the OB at 21 dpe in the GL. (**E**) The restriction of the expression pattern of Sp8 from neuroblasts to mature OB neurons originating from the medial SVZ is summarized in graph (**F**). (**G**–**I**) Representative immunostainings reveal differential Sp8 expression by the 3 main subpopulations of PG interneurons (CalR, CalB and TH) at 21 dpe. (**J**) Quantification of Sp8 expression in distinct subtypes of PG interneurons. Error bars represent the standard error of the mean; *p ≤ 0.05; **p ≤ 0.01; ***p ≤ 0.001; ****p ≤ 0.0001 determined by unpaired t-test. Scale bars: 200 µm in (**B**) 100 µM in (**B**b) 50 µm in (**A**,**B**a) 20 µm in (**A**b,**C**,**G**–**I**) 10 µm (**A**a). Dpe: days post-electroporation; RMS: Rostral Migratory Stream; GL: Glomerular layer; EPO: electroporation

Sp8 is expressed in the forebrain early during embryonic development. Its expression persists after birth in restricted regions, including the SVZ, RMS and OB. While Sp8 mRNA is detected in NSCs and transient amplifying progenitors (TAPs) of the postnatal SVZ (See Supplementary Fig. S1), nuclear expression of Sp8 becomes prominent in Dcx+ neuroblasts in the SVZ of early postnatal mice (P6) (Fig. 1A). In the OB at P23, most Sp8+ cells are observed in the GL, while some cells are also present in the granule cell layer (GCL) (Fig. 1B).

In order to investigate the pattern of Sp8 expression in distinct interneuron lineages that originate from different SVZ microdomains, we electroporated a GFP expression plasmid in lateral, dorsal and medial SVZ NSCs. Immunodetection of Sp8 in GFP+ cells in the RMS 2 days post-electroporation (2 dpe) revealed that most migrating neuroblasts express Sp8 independently of their origin (lateral EPO: 82.1% ± 3.9; n = 3 mice; dorsal EPO: 69.9% ± 3.4; n = 4 mice; medial EPO: 75.4% ± 0.7; n = 3 mice; Fig. 1C,D). This homogeneous Sp8 expression becomes gradually restricted to define subpopulations of mature neurons within the glomerular and granular cell layers. Thus, Sp8 expression is largely downregulated at 21 dpe in interneurons originating from the lateral SVZ (12.8% ± 5.8; n = 4 mice), and to a lesser extent in those originating from the dorsal SVZ (42.1% ± 5.1; n = 4 mice). In striking contrast, it is largely maintained in interneurons originating in the medial SVZ (69.6% ± 3.2; n = 4 mice) (Fig. 1E,F).

Owing to the different spatial origin of OB interneuron subtypes, these observations suggest that Sp8 expression is only maintained in specific OB lineages. We next identified the three main subtypes of PG GABAergic interneurons by immunodetection of CalB, CalR and TH markers (Fig. 1G–I). In agreement with their respective origin^5^, the vast majority of CalR+ PG interneurons expressed Sp8 (74.3% ± 2.2; n = 4 mice), while only 10.3% ± 1.7 and 18.5% ± 2.5 of CalB+ and TH+ interneurons kept expressing Sp8, respectively (n = 4 mice each) (Fig. 1J). These results were confirmed by optical density measurement of Sp8 expression in 900 randomly selected periglomerular interneurons expressing CalR, TH or CalB (n = 4 mice each; Fig. S2). Our results confirmed an absence of expression in CalB+ cells, while a weak expression was observed in some TH+ cells which was however consistently lower than in CalR+ interneurons, as previously reported^9^.

Altogether, our results highlight a complex spatial and temporal pattern of Sp8 expression. While it is expressed in migrating neuroblasts of all lineages, its expression becomes gradually restricted to CalR+ PG interneurons, suggesting a dual role in OB neurogenesis.

### Sp8 influences proliferation and cell cycle exit in the postnatal SVZ

To investigate the early role of Sp8 in SVZ neural stem cells and their immediate progeny, we used an electroporation approach to transiently manipulate its expression. Gain- and loss-of-function experiments were performed with non-integrative plasmids coding for the Sp8 protein or a shRNA against Sp8 (see Supplementary Fig. S3), respectively.

**Figure 2:**
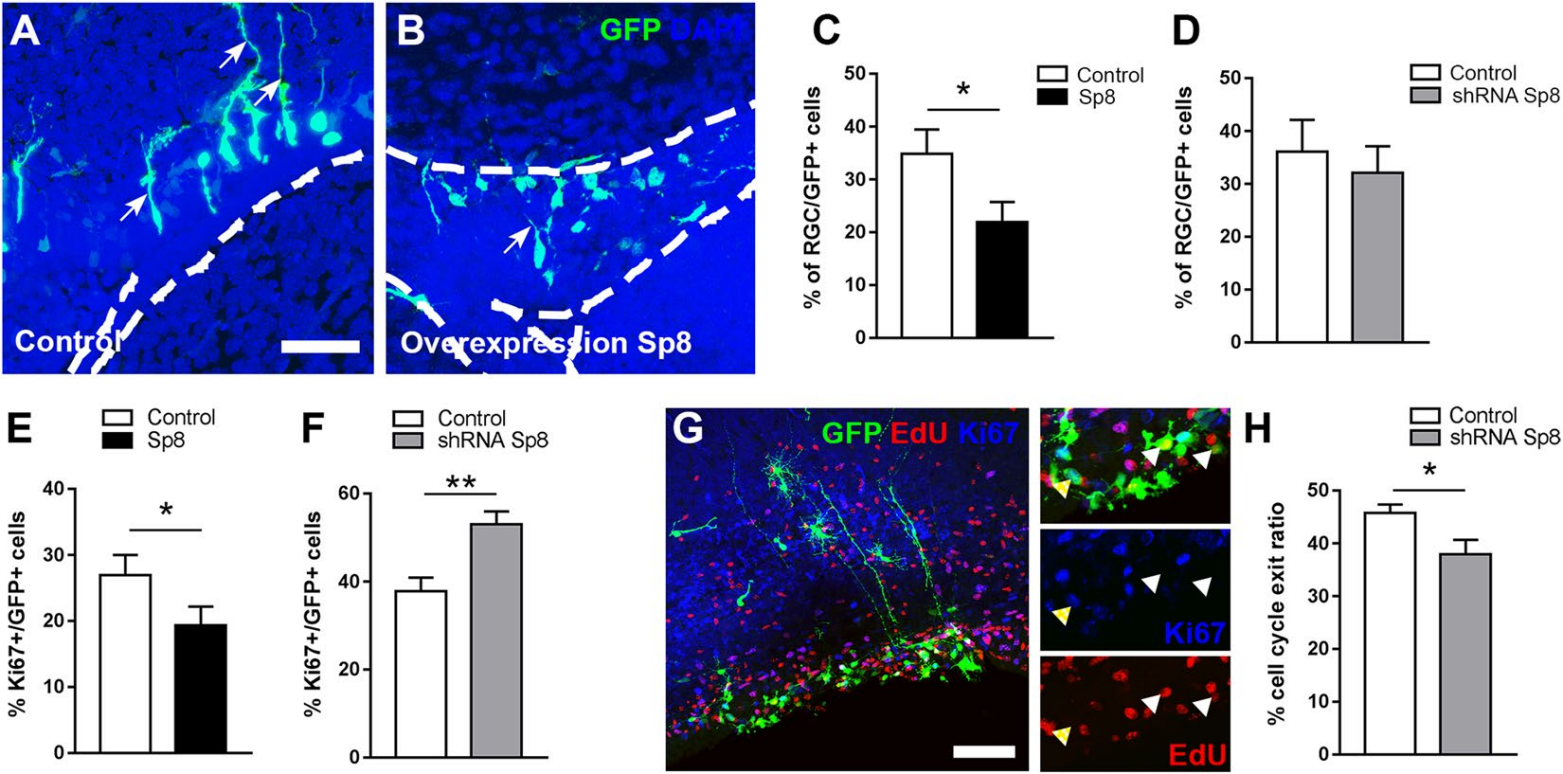
Transient manipulation of Sp8 expression in NSCs influences proliferation in the postnatal SVZ. (**A**,**B**) Confocal pictures revealing the morphology of labelled progenitors in the dorsal SVZ 4 days after overexpression of Sp8. Scale bar: 40 µm. (**C**,**D**) Quantification of the proportion of RGCs after transient Sp8 overexpression (**C**) and deletion (**D**) in the dorsal and lateral SVZ. (**E**,**F**) Quantification of the percentage of proliferative cells (Ki67+) following Sp8 overexpression (**E**) and deletion (**F**) in the dorsal and lateral SVZ. (**G**) Representative immunostaining of proliferative labelled progenitors (GFP+ Ki67+, yellow arrowheads) at 4 dpe in the dorsal SVZ. Animals were injected with a single dose of EdU 24 h before sacrifice, to identify the population that exit the cell cycle between 3 and 4 dpe (i.e. EdU+/Ki67−, white arrowheads). High magnification on Ki67+ and EdU+ progenitors are shown on the right panels. Scale bar: 50 µm. (**H**) The percentage of progenitors that exit the cell cycle between 3 and 4 dpe is determined by the quantification of EdU+ Ki67−/EdU+ cells in dorsal and lateral SVZ. Error bars represent the standard error of the mean; *p ≤ 0.05; **p ≤ 0.01 determined by Mann Whitney test (**D**,**E**,**H**) or unpaired t-test (**C**,**F**). Abbreviations: RGCs: Radial Glia Cells.

Early after birth, NSCs and transient amplifying progenitors can be readily identified based on their morphology^10^. While NSCs present a radial glia cells (RGCs) morphology, progenitors appear as round cells without processes. Following Sp8 overexpression in the postnatal SVZ, we observed a reduction in the proportion of RGCs within the population of GFP+ electroporated cells in the SVZ at 4 dpe (Control: 34.8% ± 4.7; n = 12 mice; Sp8: 21.9% ± 3.8; n = 14 mice; Fig. 2C). In agreement with the absence of Sp8 expression in most RGCs and progenitors (see above), Sp8 knockdown showed no effects onto RGCs numbers (Control: 36.1% ± 6; n = 12 mice; shRNA Sp8: 32.1% ± 5.1; n = 15 mice; Fig. 2D). These data suggests that forced Sp8 expression in RGCs promotes their premature differentiation.

We next investigated the potential role of Sp8 in controlling proliferation and cell cycle exit. We analysed the proliferative capacities of GFP+ electroporated cells (RGCs and TAPs) following transient overexpression and knockdown of Sp8. Sp8 overexpression led to a significant decrease in the proportion of proliferative cells in the SVZ at 4 dpe (Control: 26.95% ± 3.0; Sp8: 19.33% ± 2.8, n = 8 mice each; Fig. 2E). In contrast, Sp8 knockdown resulted in an increased proliferation, suggesting a reduced cell cycle exit of late progenitors (Control: 37.87% ± 3.0; n = 8 mice; shRNA Sp8: 53.03% ± 2.9, n = 10 mice; Fig. 2F). Indeed, analysis of the cell cycle exit by injection of EdU 24 h before analysis and co-localization with the proliferation marker Ki67 progenitors (Fig. 2G), revealed a reduced number of EdU+/Ki67− electroporated cells within the SVZ (Control: 45.75% ± 1.6; n = 8 mice; shRNA Sp8: 37.96% ± 2.7, n = 10 mice; Fig. 2H). Altogether, these results reveal that Sp8 promotes cell cycle exit and thereby promotes the differentiation of postnatal progenitors.

### Sp8 is not involved in neuroblasts migration nor CalR+ interneurons specification

**Figure 3:**
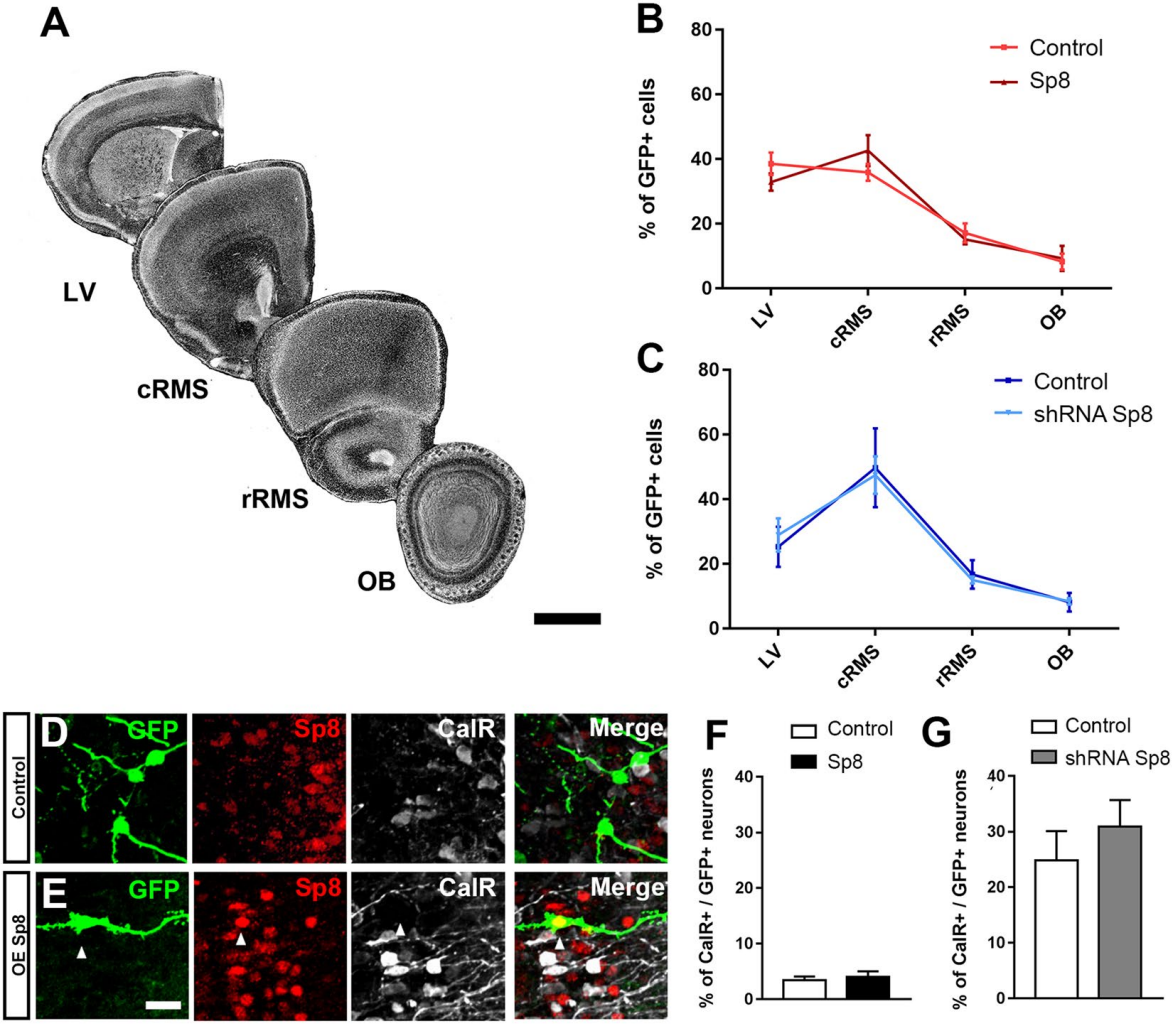
Transient manipulation of Sp8 expression does not influence neuroblasts migration nor their specification in CalR+ PG interneurons. (**A**) Illustration of coronal sections at distinct rostro-caudal coordinates counterstained with DAPI. Scale bar: 500 µm. (**B**,**C**) Quantification of the percentage of GFP+ cells distributed in the lateral ventricle, in the caudal and rostral RMS and in the OB at 4 dpe. (**D**,**E**) Representative immunostaining of GFP+ newborn neurons expressing CalR at 21 dpe after overexpression. Scale bar: 10 µm. (**F**,**G**) Quantification of the proportion of CalR+ interneurons in the granular and glomerular cell layers 21 days after Sp8 overexpression or deletion in the lateral or medial SVZ, respectively. Error bars represent the standard error of the mean; significance tested by unpaired t-test. LV: lateral ventricle; cRMS: caudal part of the rostral migratory stream; rRMS: rostral part of the rostral migratory stream; OB: olfactory bulb.

The nuclear expression of Sp8 in Dcx+ cells questions its role in neuroblasts migration. We quantified the number of electroporated cells in the SVZ as well as at different rostro-caudal coordinates of the RMS, following overexpression or knockdown of Sp8 as previous^11^. At 4 dpe, most of the electroporated cells were observed within the RMS caudal more regions, with only few having reached its rostral most compartment, corresponding to the OB peduncle (Fig. 3A–C). Sp8 overexpression (Fig. 3B) or knock down (Fig. 3C) had no effect on this distribution, indicating that Sp8 is not involved in the control of neuroblast migration.

We next investigated the role of Sp8 in CalR+ interneurons specification. We took advantage of the regional generation of CalR+ PG interneurons subtypes in the postnatal SVZ. An instructive role of Sp8 in CalR+ interneurons specification was investigated by overexpressing it in the lateral SVZ, which only produces rare CalR+ PG interneurons^5^. Among all interneurons produced by this microdomain, only 3.4% ± 0.7 (n = 5 mice) expressed CalR (Fig. 3F). This percentage was not changed 21 days following Sp8 overexpression (4% ± 1; n = 5 mice), despite persistent immunodetection of Sp8 in electroporated cells (Fig. 3E). We next investigated the consequences of Sp8 knockdown in CalR+ interneuron specification by electroporating a shRNA construct in the medial SVZ. As many as 24.86% ± 5.2 of the electroporated GFP+ cells originating from this wall and migrating in the glomerular and granular cell layers acquire expression of CalR at 21 dpe, a percentage that remained unchanged by Sp8 knockdown (30.91% ± 4.8; n = 4 mice; Fig. 3G). Together, these results suggest that Sp8 is neither necessary nor sufficient to specify CalR+ interneurons in the postnatal OB.

### Early Sp8 deletion affects newborn neurons survival and maturation

**Figure 4:**
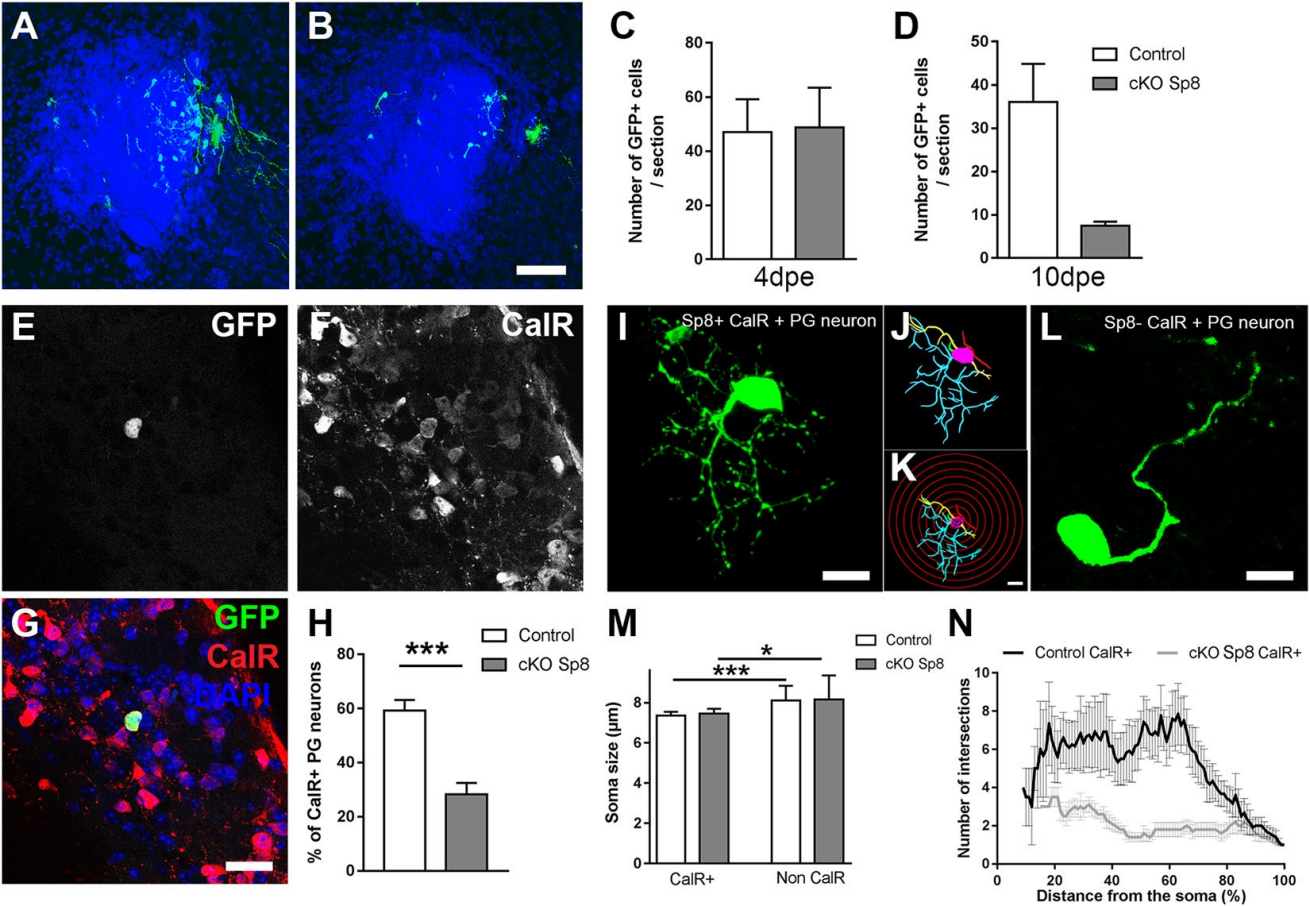
Deletion of Sp8 in the medial SVZ affects the maturation and survival of CalR+ PG interneurons. (**A**,**B**) Illustration of GFP+ migrating neuroblasts in the RMS in control and cKO Sp8 brains at 10 days post medial EPO. Scale bar: 200 µm. (**C**,**D**) Quantification of GFP+ neuroblasts in the RMS at 4 dpe (**C**) and 10 dpe (**D**) after permanent Sp8 deletion in medial NSCs. (**E**,**G**) Immunostaining for GFP+ labelled neurons expressing CalR in the GL. Scale bar: 20 µm. (**H**) Quantification of the proportion of GFP+/CalR+ in control and following Sp8 deletion. (**I**–**L**) Representative confocal picture of CalR+/Sp8+ (**I**) and CalR+/Sp8− PG interneurons (**L**). Scale bars: 10 µm. (**J**,**K**) 3D reconstruction of the CalR+ interneuron shown in I with the software Neurolucida 360 (**J**). A sholl analysis was performed by counting the number of dendrite intersections with concentric circles separated from each other by 5 µm radius (**K**, see also panel N). (**M**) Measurement of CalR+ and non-CalR+ PG interneurons soma size in controls and following permanent Sp8 deletion. (**N**) Sholl analysis of control and Sp8 cKO CalR+ interneurons. Error bars represent the standard error of the mean; *p ≤ 0.05; **p ≤ 0.01; ***p ≤ 0.001 determined by unpaired t-test.

In order to perform a permanent deletion of Sp8, we used floxed Sp8 (Sp8^fl/fl^) transgenic mice. We induced early and permanent Sp8 deletion in NSCs and their progeny by electroporating a plasmid coding for the Cre-recombinase in select SVZ walls. The conditional and permanent deletion of Sp8 (cKO) was confirmed at 21 dpe in periglomerular neurons originating from the medial SVZ as revealed by the complete absence of Sp8 expression in electroporated GFP+ cells (See Supplementary Fig. S4). Electroporation of NSCs of the medial SVZ revealed a delayed loss of labelled neuroblasts, which was not visible at 4 dpe but became prominent at 10 dpe (Fig. 4A–D). In order to investigate if these effects were specific of the CalR+ lineage, we quantified the proportion of GFP+ PG interneurons expressing CalR at 21 dpe. As expected, CalR+ interneurons represent 59.2% ± 3.9 (n = 4 mice) of GFP+ PG interneurons in control brains, while they only represent 28.2% ± 4.2 (n = 4 mice) following Sp8 ablation (Fig. 4H). The other PG interneurons subtypes (i.e. CalB+ and TH+ interneurons) were also affected although to a lesser extent following permanent deletion of Sp8 in the dorsolateral SVZ (data not shown). Among the few CalR+ surviving neurons, the majority showed an atrophied dendritic arborization compared to nearby Sp8 expressing neurons (Fig. 4I–L), although the soma size remained unchanged (Fig. 4M). A sholl analysis indeed revealed a consistent reduction of the complexity of their arborization when compared to Sp8-expressing CalR+ PG interneurons (Fig. 4N). Altogether, these results support a role of Sp8 as a terminal selector gene that insure the correct maturation and survival of maturing CalR+ interneurons.

### Late Sp8 ablation supports a specific role in CalR+ interneurons survival

**Figure 5:**
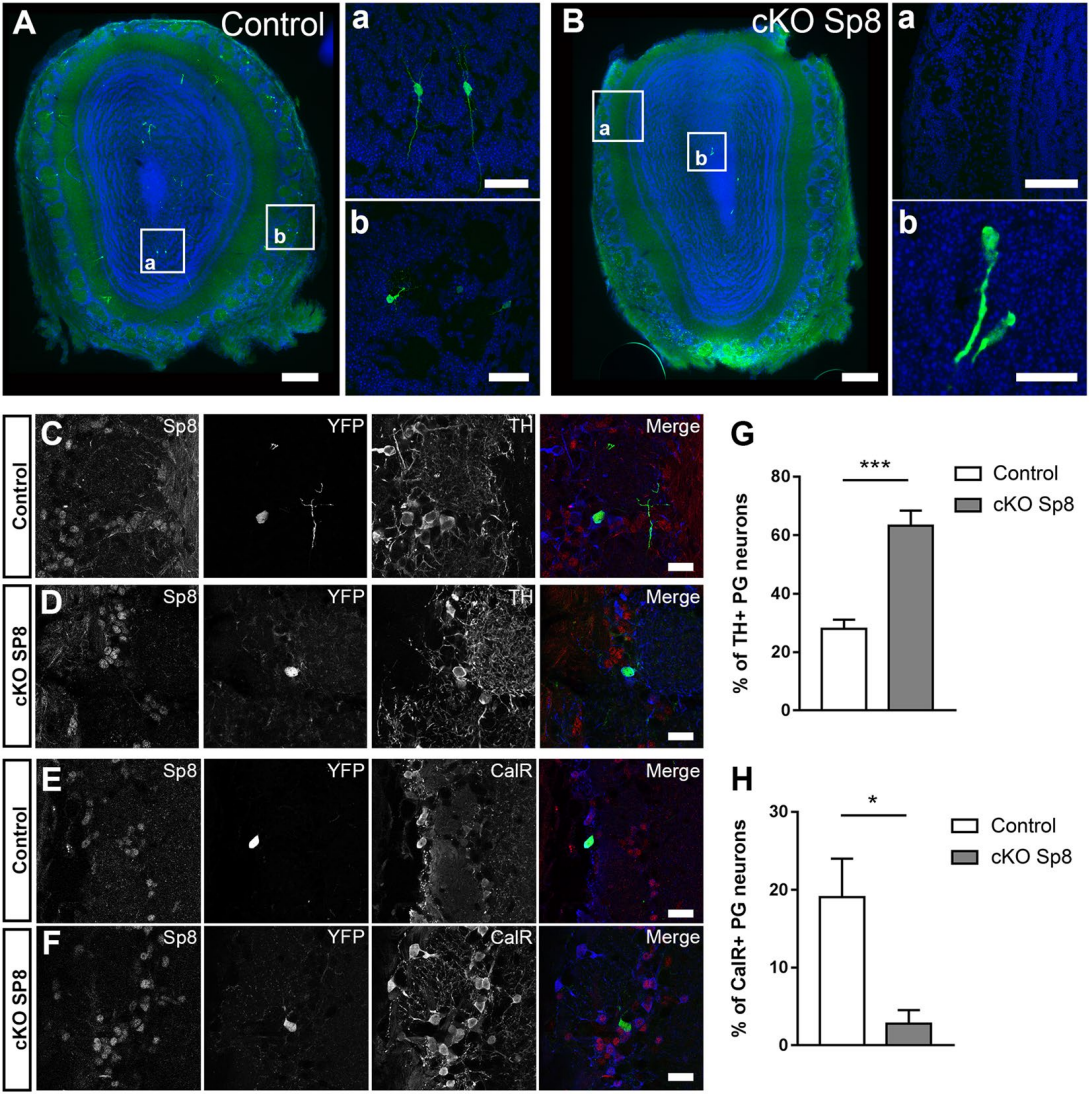
Delayed deletion of Sp8 in maturing neurons leads to a severe and specific loss of CalR+ PG interneurons. (**A**,**B**) Illustration of labelled neurons in the RMS, GL and GCL of the OB at 21 dpe in control mice (**A**) or following Sp8 deletion at 11 dpe (**B**). Scale bars: 200 µm, 20 µm (**A**a) 40 µm (**A**b) 50 µm (**B**a) 2 µm (**B**b). (**C**–**F**) Immunostainings revealing TH+ and CalR+ expression in GFP+ neurons in control brains or following Sp8 deletion. Scale bars: 20 µm (**G**,**H**) Quantification of the proportion of CalR+ and TH+ among GFP+ PG interneurons in controls and following Sp8 deletion. Error bars represent the standard error of the mean; *p ≤ 0.05; ***p ≤ 0.001 determined by Mann Whitney test (CalR+) or unpaired t-test (TH+).

In order to confirm the importance of Sp8 expression in PG CalR+ interneurons maturation and survival, we induced Sp8 ablation at later stages of newborn neurons differentiation. An inducible Cre recombinase (i.e. ERT2-CRE-ERT2) was electroporated in the medial SVZ of Sp8^fl/fl^ transgenic mice crossed with Cre reporter mice (RosaYFP) and Sp8 deletion was induced at 11 dpe by tamoxifen injection. Interestingly, the effect of delayed Sp8 ablation on CalR+ interneurons survival was similar but more drastic than following early Sp8 deletion. Indeed, no labelled newborn neurons were detected in the GL following Sp8 deletion (Fig. 5A,B). However, some migrating neuroblasts were still observed in the core of the OB (Fig. 5B) which confirmed the efficiency of the electroporation and further supported that early stages of neuronal differentiation, including migration were not affected.

In order to assess if these effects were specific to CalR+ neurons, we performed a targeted ablation in the dorsolateral SVZ which generate both TH+ and CalR+ PG interneurons. As expected, the population of CalR+ PG interneurons was lost following Sp8 deletion (19.1% ± 5.0 vs 2.8% ± 1.8. Figure 5H). Simultaneously, the proportion of TH+ interneurons, the main interneuron subtype produced by the dorsolateral SVZ increased proportionally (28.0% ± 3.1 vs 63.2% ± 5.1; Fig. 5G). Thus, interneuron populations that downregulate or express low levels of Sp8 in the OB are only minimally affected by Sp8 deletion.

## Discussion

Through this study, we demonstrate that Sp8 is not involved in the early specification of CalR+ PG interneurons of the OB but plays a crucial role in maintaining their integrity.

Our observations emphasize a complex expression pattern of Sp8 during postnatal OB neurogenesis, which suggests distinct functions at defined stages of the neuronal differentiation process. Previous studies have demonstrated a strict spatial origin of OB interneurons subtypes, which is particularly apparent for PG interneurons subtypes (reviewed in^1^). Thus, CalR+ interneurons originate from both the medial and dorsal walls of the SVZ, while TH+ and CalB+ arise from the dorsal and lateral walls respectively. This spatial origin of defined neuronal lineages can be resolved by targeted electroporation of distinct SVZ walls^5^. Fate mapping of these distinct microdomains reveals that Sp8 expression is initially common to all migrating neuroblasts in the postnatal RMS. Interestingly, Sp8 transcripts can be detected in NSCs and TAPs but expression of the protein only appears in Dcx+ neuroblasts of the SVZ and in the RMS. This early priming has been previously reported for adult NSCs, particularly for neurogenic TFs (see for example^8,12^).

Gain- and loss-of-function experiments suggest an early role for Sp8 in controlling cell cycle exit and initiation of neuronal differentiation. Thus, ectopic Sp8 expression in NSCs promotes the premature differentiation of progenitors into neuroblasts and depletes the pool of RGCs. In contrast, Sp8 knock down does not affect NSCs proliferation, in accordance with the lack of protein expression in these cells, but decreases cell cycle exit of postnatal progenitors. These results are in line with the previously described role of Sp8 in regulating proliferation and differentiation processes during embryogenesis^13,14^. The molecular mechanisms underlying these effects need further investigations. Sp8 is known to tightly control proliferation and differentiation during embryonic brain development^13,15,16^, possibly by activating and sustaining Fgf8 signalling in the rostral most forebrain regions^14,16^. A study published by Li *et al*. recently confirmed the pro-neurogenic effect of Sp8 in the adult SVZ coordinately with Sp9^17^. They identified Prokr2, a G protein-coupled receptor of chemokine Prok2 and Tshz1 (Teashirt Zinc Finger Homeobox 1), as a downstream target of Sp8/9. This receptor is expressed in newly born neuroblasts and is required for neuronal differentiation. Thus, Sp8 appears as a key factor influencing major signalling pathways to regulate neuronal differentiation progression in the postnatal brain.

As neurons mature, our results show that Sp8 expression becomes restricted to CalR+ PG interneurons, in line with previous findings^7,9^. The early expression of Sp8 in neuroblasts of all lineages however indicates that it cannot be used as a reliable marker of CalR+ fated neuroblasts, as previously suggested^18^. Interestingly, Sp8 deletion affects CalR+ PG interneurons numbers, while other interneurons subtypes are only minimally affected. This observation may be explained by a role of Sp8 in CalR+ PG interneurons specification, and/or survival. A role of Sp8 in specifying CalR+ PG interneurons is not supported by our results. Indeed, overexpression of Sp8 in NSCs of the lateral SVZ was not sufficient to generate CalR+ PG interneurons. Inversely, Sp8 knockdown in NSCs of the medial SVZ does not impact the generation of CalR+ PG interneurons, further supporting that this transcription factor is not required for the specification of this interneuron population. The specification of CalR+ PG interneurons may rather involve other TFs such as Zic1 and Zic2 which have recently been reported to induce the generation of CalR+ interneurons while suppressing the dopaminergic fate of dorsal NSCs^6^. In contrast, our results support a role of Sp8 in promoting CalR+ PG interneurons maturation and survival. Interestingly, the apoptotic phenotype in CalR+ neurons appears to be stronger after delayed Sp8 deletion in migrating neuroblasts while TH+ interneurons were not affected, as evidenced by the increase of their relative proportion. A similar survival effect was recently reported in Sp8/Sp9 cKO mice^17^. However, the observed cell death also encompassed TH+ neurons suggesting a broad and partially non-overlapping expression of Sp8 and Sp9 in mature PG interneurons subtypes. The mechanisms involved in the survival of mature CalR+ interneurons remain to be elucidated. This apoptosis is likely to not occur abruptly. Indeed, activated caspase 3 immunodetection did not reveal any peak of synchronized apoptosis at 4 or 10 days following Sp8 depletion. As previously observed in other contexts, apoptosis is likely to occur over extended periods of time and may therefore be difficult to detect at a single time point^19^. This goes in line with the detection of surviving cells with atrophied morphologies in our experiments. The few CalR+ interneurons that persist after Sp8 ablation exhibit an atrophied arborization, which support a default in maturation and integration of these PG interneurons.

Our observations all converge in defining Sp8 as a terminal selector gene (TSG) in CalR+ PG interneurons. TSGs are TFs that specify and maintain the identity of mature neuron subtypes and insure their maturation and survival. They maintain individual neuronal identities by directly controlling the expression of downstream, terminal differentiation genes. For instance, TSGs maintain the neuronal identity by activating neurotransmitter-type gene batteries encoding the capacity for their synthesis, reuptake, and plasma or vesicular membrane transport^20–22^. Other studies also provide intriguing examples of their requirement for maintenance of neuronal connectivity, such as for Nurr1 in dopaminergic neurons of the OB^23^. In addition, TSGs are continuously required for survival of post-mitotic neurons through transcriptional maintenance of proper levels of expression of several anti-apoptotic and pro-apoptotic genes^24^. Although the exact mechanisms by which Sp8 acts remain to be explore, other studies have identified TSGs in the OB as well as in other brain regions, which are likely to act in similar ways. For instance, Pax6 have been suggested to play a role both in the early specification of TH+ interneurons but also in their survival once mature in the OB^25^. Pax6 modulates apoptotic signalling pathway by regulating the expression of the crystallin αA which prevent caspase 3A activation. Experiments aimed at measuring and possibly rescuing crystallin αA level of expression following Sp8 deletion will be necessary to investigate if similar mechanisms are in play to mediate CalR+ PG interneuron survival.

Our observation of a tight control of CalR+ PG interneuron survival suggests the existence of multiple mechanisms, acting at distinct stages of the neurogenesis process as well as onto different lineages, to control the number of newborn neurons incorporating in the OB circuitry. Such a fine control of the production and maintenance would be necessary if defined subtypes of PG interneurons would control different aspects in the treatment of olfactory signal integration. Indeed, the specific properties and distinct connectivity of PG interneurons subtypes^26^ suggest different roles in the treatment of olfactory information^3,26–28^. A tight control of PG interneurons numbers might be key in processing olfaction signal, which remains to be functionally explored. In this context, TFs such as Sp8, acting as a TSG, would allow the number of interneurons of defined subtype to be dynamically and finely controlled in order to maintain a functional network.

## Materials and Methods

### Animals

Transient genetic manipulation were performed in CD1 mice (Charles Rivers). Permanent deletion of Sp8 were assessed in Sp8^fl/fl^ (Strain name: Sp8^tm2Smb^, Jackson Laboratory, Bar Harbor, ME, USA). These mice were crossed with R26R-EYFP mice (Strain name: B6.129X1-Gt(ROSA)26Sortm1(EYFP)Cos/J; Jackson Laboratory) to follow recombined cells thanks to EYFP expression. The resulted RosaYFP-Sp8^fl/fl^ mice were kindly donated by D^r^ Ugo Borello. All procedures were performed in accordance with European requirements 2010/63/UE and have been approved by the Animal Care and Use Committee CELYNE (APAFIS#187 & 188). Animal procedures were executed in accordance with Swiss/French law, with strict consideration given to the care and use of animals

### Plasmids

#### Subcloning of Sp8

The mouse *Sp8* gene was amplified from a pXY-Asc plasmid (clone ID 30653287, Open Biosystems, Huntsville, USA) by PCR using specific primers designed to add Xho I and Sac I restriction sites (sense primer (Xho5′) 5′ agt cct cga gat ggc aac ttc act tct ag 3′; antisense primer Sac 3′ gat cga gct ctc act cca ggc cgt tgc g). The PCR product was purified from an agarose gel using a Qiagen kit following manufacturer’s instructions and digested with the corresponding restriction enzymes (XhoI (C^TCGAG) #R0146S; SacI (GAGCT^C) #R0156S) before subcloning it into a pCAAG-IRES-nlsGFP plasmid. The correct integration of the gene was verified by sequencing. To check the efficient expression of the generated plasmid, about 100.000 HEK cells were transfected with 2 µg of DNA (Sp8-IRES-nlsGFP plasmid alone (called Sp8 plasmid in this study to simplify) or pCAAG+ pCX-GFP plasmid used as a positive control) using Lipofectamine prepared in Optimem (See Supplementary Fig. S3).

#### shRNA testing efficiency

Five different shRNAs against Sp8 were purchased from Open Biosystems (Huntsville, USA) (TRCN0000085558, TRCN0000085559, TRCN0000085560, TRCN0000085561, and TRCN000008555862). Their interfering efficiency was first tested *in vitro*. HEK cells were co-transfected with shRNA + pCAAG-Sp8-IRES-nlsGFP plasmids using the lipofectamine method following manufacturer’s instructions (Thermo Fisher Scientific, Waltham, MA USA). 72 h post transfection, cells were harvested and lysed to extract RNA (Qiagen RNAeasy extraction kit, Hilden, Germany), which was then retro-transcripted into cDNA to be analysed by qPCR using a 1:100 dilution. The standard curve was prepared from a Sp8 overexpressing sample that was diluted at different concentrations. *HPGRT* was used as housekeeping gene. The shRNA 9 showing the best inhibition was chosen for further experiments (See Supplementary Fig. S2).

### Electroporations

CD1, Sp8^fl/fl^ or RosaYFP-Sp8^fl/fl^ pups were electroporated at 2 postnatal days (P2) as described previously (Boutin *et al*. 2008; Fernandez *et al*. 2011). Briefly, pups were anesthetized in ice and placed on a custom made support in a stereotaxic rig. Injections were performed at the midpoint of a virtual line traced between the eye and lambda. A 34 G needle attached to a Hamilton syringe was inserted at a depth of 2 mm from the skull surface and 1.5 µl of plasmid solution was injected into the lateral ventricle.

For fate mapping experiments, a solution of pCX-GFP [5 µg/µl] plasmid was used in CD1 mice. For gain- and loss–of-function experiments, Sp8 or shRNA plasmids were combined with pCX-GFP plasmid at a 3:1 ratio [5 µg/µl] in CD1 mice. For early and late conditional deletion experiments, pCAG-CRE or pCAG-ERT2-CRE-ERT2 plasmids was combined to pCX-GFP plasmid at a 2:1 ratio [5 µg/µL] in Sp8^fl/fl^ mice. Co-electroporation of both plasmids resulted in co-expression in 80% of targeted cells as previously shown (Boutin *et al*. 2008). For late conditional deletion, pCAG-ERT2-CRE-ERT2 plasmid was used in RosaYFP-Sp8^fl/fl^ mice. Empty or scrambled plasmids +/−pCX-GFP were used as controls in littermates.

A contrast agent (fast green solution (0.2%) in sterile PBS) was added to the plasmid solution to confirm the accuracy of intraventricular injections. Successfully injected mice were then subjected to 5 electrical pulses (95 V, 50 ms, separated by 950 ms intervals) using the Super Electroporator NEPA21 type II (Nepa Gene Co., Ltd, Ichikawa-City, Chiba, Japan) and tweezer electrodes coated with conductive gel (Signa gel, Parker Laboratories, Fairfield, New Jersey, USA). Electrodes were positioned to target either the lateral or the medial wall as previously described (Fernandez *et al*. 2011). After electroporation pups were warmed up until full recovery from the anaesthesia and returned to their mother.

### EdU injections

EdU (Life technologies, Carlsbad, California, USA) was diluted in sterile PBS at 10 mg/mL and injected i.p. at a final concentration of 50 mg/kg.

### Tissue processing

Mice were sacrificed at 4 days post electroporation (dpe) or 21 dpe to assess the effect on radial glia or mature neurons, respectively. After terminal anaesthesia with an intraperitoneal overdose of pentobarbital (60 mg/kg), mice were transcardially perfused with a ringer solution, followed by 4% paraformaldehyde solution in 0.1 M phosphate buffer (PB, prepared with Sodium phosphate dibasic anhydrous and Sodium Phosphate dibasic dihydrate diluted in distilled water; Sigma-Aldrich, Saint-Louis, Missouri, USA). Brains were removed, post-fixed for two days in the same fixature and cut with a vibratome at 50 µm (VT1000 S, Leica Biosystems, Wetzlar, Germany). Coronal sections were collected from the OB to the lateral ventricle in series of 6 and kept at −20 °C in antifreeze solution (15% glucose, 0,02% sodium azide (sigma), 30% ethylene glycol, 0,1 M PB; Sigma-Aldrich, Saint-Louis, Missouri, USA).

### Immunohistochemistry

Free floating sections or cells grown on coverslips are blocked in TNB-Tx buffer (0.25% BSA, 0.05% Casein, 0.25% Top block +0.4% triton X100; Sigma-Aldrich, Saint-Louis, Missouri, USA). When antigen retrieval was needed, sections were incubated at 80 °C in Citrate Buffer (10 mM, pH 6) for 20 minutes. Sections are then incubated with primary antibodies at 4 °C overnight. The following antibodies were used in the present study: anti-Sp8 (Rabbit polyclonal, 1:2000, Millipore, Massachusetts, USA); anti-calretinin (Rabbit polyclonal or mouse monoclonal, 1:2000, SWANT, Marly, Fribourg, Switzerland); anti-calbindin (Mouse monoclonal, 1:2000, SWANT, Marly, Fribourg, Switzerland); anti-tyrosine-hydroxylase (Mouse monoclonal, 1:400, Millipore, Massachusetts, USA); anti-GFP (Chicken polyclonal, 1:1000, AVES Lab, Tigard, Oregon, USA); anti-Ki67 (Mouse monoclonal, 1:400, Abcam, Cambridge, County of Cambridgeshire, UK); anti-Ki67 (Rabbit monoclonal, 1:500, Thermo Fisher Scientific, Massachusetts, USA), anti-NeuN (Mouse monoclonal, 1:400, Millipore, Billerica, Massachusetts, USA); anti-Doublecortin (Goat polyclonal, 1:500, Santa Cruz Biotechnology, California, USA); anti-Caspase (Rabbit polyclonal, 1:1000, Millipore, Massachusetts, USA). Following extensive washing in TNB-Tx, sections were incubated with the corresponding secondary antibody (1:1000, all from Thermo Fisher Scientific, Massachusetts, USA). To amplify the signal in YFP cells, sections were incubated with anti-GFP primary antibody followed by a biotinylated anti-chicken secondary antibody (Donkey, 1:1000, Jackson labs technologies, Nevada, USA) and incubated in streptavidin-DTAF (1:250, Jackson labs technologies, Nevada, USA) for 30 min. Finally, sections were counterstained with DAPI and mounted with Fluoromount mounting medium.

### Acquisitions and quantifications

Quantifications were performed on pictures taken with a Leica TCS SPE II confocal microscope and a TCS SP5 confocal microscope (Leica, Wetzlar, Germany) equipped with a 40× objective (NA 1.25) and processed with Image J and Photoshop CS4. All quantifications were performed on raw images, except for optical densitometry (OD) analysis, where grey value measurements were performed on confocal images processed by Image J. Stack images were flattened to obtain stacks of 8–10 µm maximum to avoid cells superpositions. Sp8 channel stacks were not processed. Cells with an OD < 25 were very faint or relatively ambiguous and were considered as negative cells for quantification on raw images. For 3D neurons reconstruction, 0.3 µm stack images were taken with a confocal microscope and 3D reconstruction were performed and analysed with the software Neurolucida 360 (MBF Bioscience, Vermont, USA). Statistical significance was determined by two-tailed unpaired *t* test at the *p* < 0.05 level or a Mann-Whitney test when data did not assume a normal distribution (tested with a Shapiro-Wilk test) (Prism 7, GraphPad Software, California, USA).

## Electronic supplementary material

Supplementary figures
