# Supplementary material for: A dual role for the transcription factor Sp8 in postnatal neurogenesis
Source: Sci Rep. 2018 Sep 28;8:14560. doi: 10.1038/s41598-018-32134-6 (PMC6162233; doi:10.1038/s41598-018-32134-6)
Supplement: Supplementary file 1 — Supplementary figures [file 41598_2018_32134_MOESM1_ESM.pdf]

A dual role for the transcription factor Sp8 in postnatal neurogenesis

Elodie Gaborieau[1]*, Anahi Hurtado-Chong[2], Maria Fernández[2], Kasum Azim[2], and Olivier Raineteau[1,2] *

SUPPLEMENTARY INFORMATION

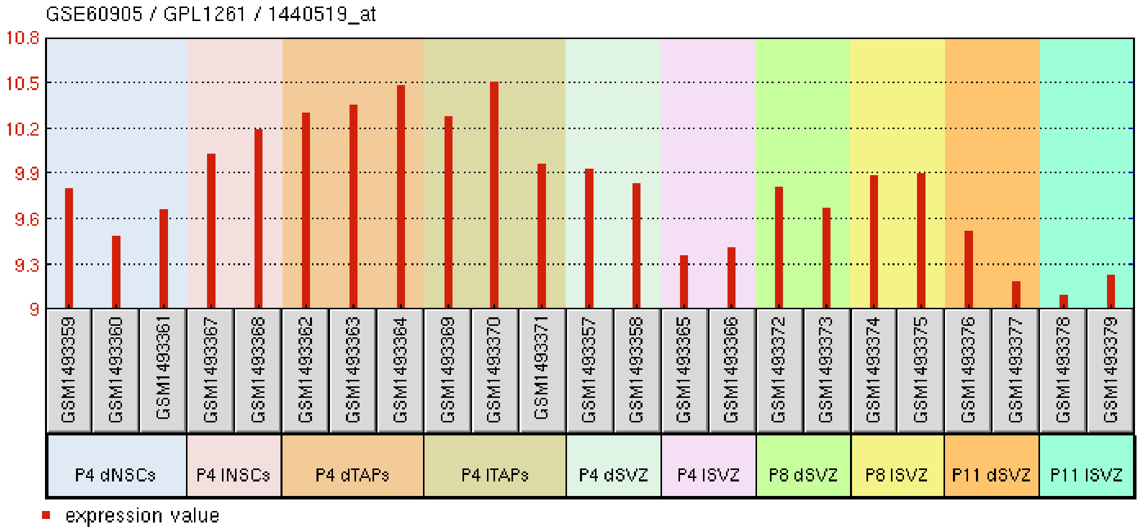

Supplementary figure S1: Detection of Sp8 mRNA in microarrays of the postnatal SVZ

To examine Sp8 transcript expression within the dorsal and lateral regions of the lateral ventricle, we made use of previously published datasets (Azim et al., 2015, GSE60905). Sp8 mRNA is detected in NSCs and TAPs of both the dorsal and lateral SVZ.

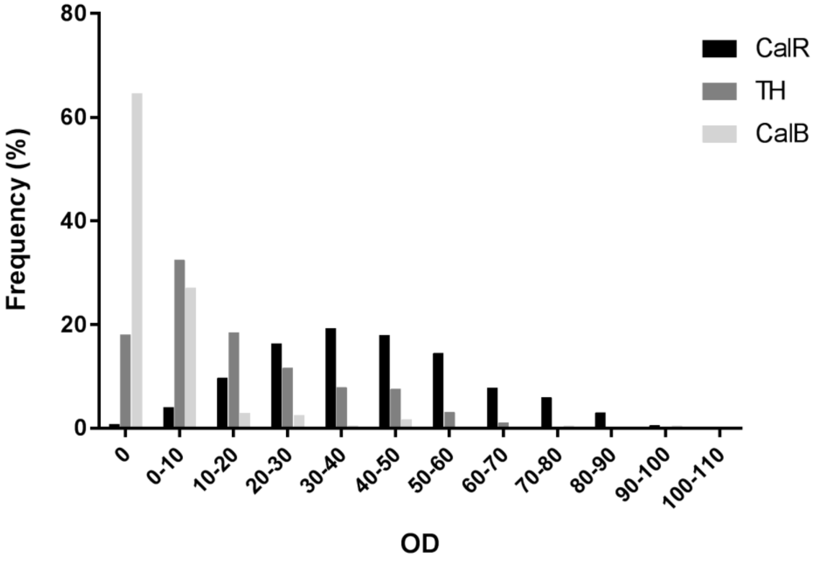

Supplementary figure S2: Optical densitometry measurement of Sp8 expression in periglomerular interneurons

The optical density of Sp8 immunostainings was measured and compared between the 3 main periglomerular interneurons subtypes, expressing CalR, CalB and TH. The average grey values of expression were close to background (i.e. 20-25) for CalB+ cells, confirming lack of Sp8 expression in this interneuron subtypes. In contrast, the vast majority of CalR+ interneurons presented averaged grey values above 30, up to 100, confirming high Sp8 expression in this interneuron subtype. Interestingly, averaged grey values of 30 to 50 were observed in a small population of TH+ interneurons, indicating that some of these interneurons express significant level of this protein. It should be noted that this expression remains nevertheless lower than in CalR+ interneurons.

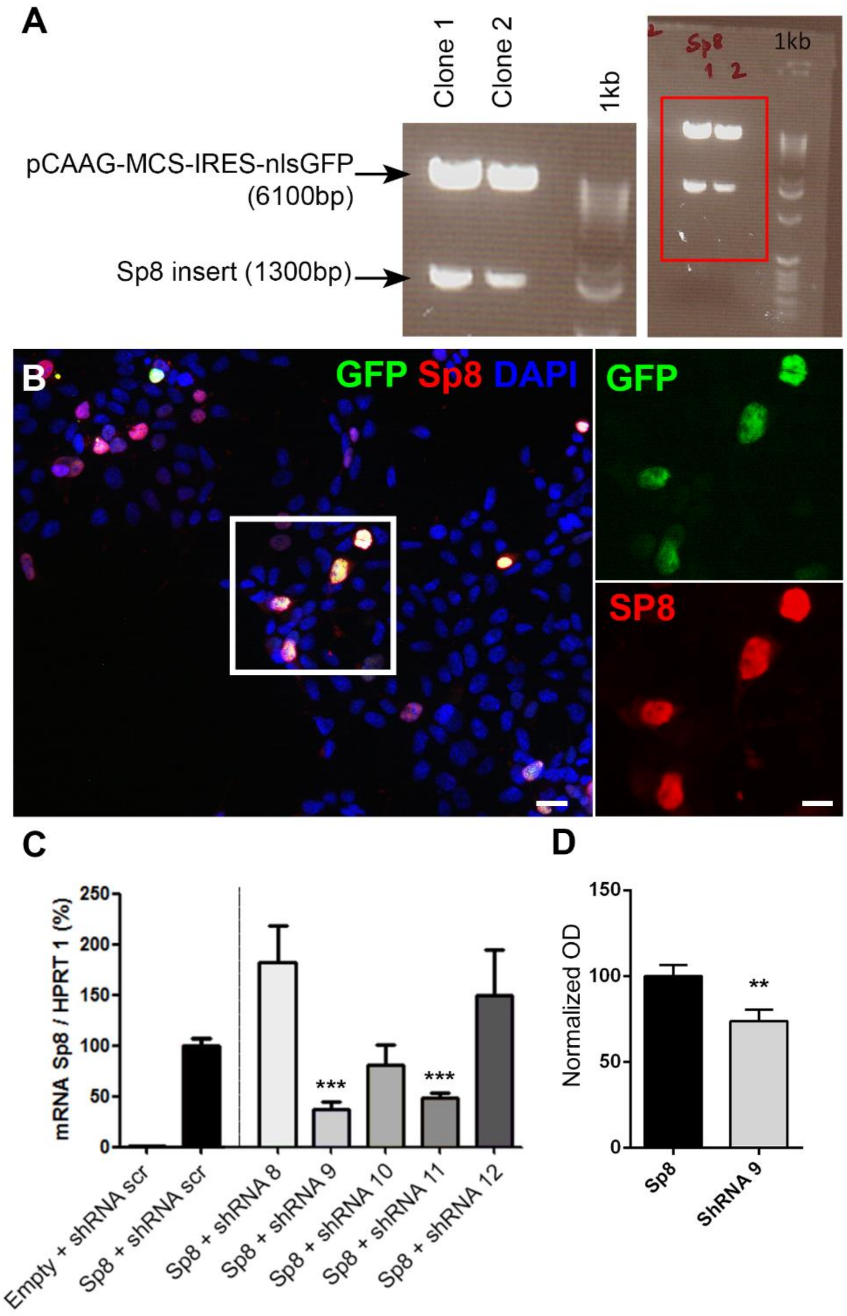

Supplementary figure S3: Validation of plasmids for SP8 overexpression and knockdown

(A) The successful cloning of Sp8 insert in the pCAAG-IRES-nlsGFP vector was verified after digestion of the PCR products with the corresponding restriction enzymes. Two different Sp8

clones were generated and digested with 5'Xho1 & 3' Sac1 restriction enzymes. The upper band reveals the presence of the pCAAG-IRES-nlsGFP vector (6100bp) and the lower band indicates the desired Sp8 gene insert (1300bp). (B) The efficiency of the Sp8 expression was validated by immunocytochemistry 48 hours after transfection of HEK cells with the pCAAG-Sp8-IRES-nlsGFP plasmid. Transfected cells expressing the GFP reporter, co-express Sp8. Non-transfected cells (counterstained with DAPI) do not express GFP nor Sp8. (C) The efficiency of five different shRNA (shRNA8 to 12) was tested on HEK cells co-transfected with the Sp8 expressing plasmid. Sp8 expression was measured by real time PCR 48 hours post-transfection and compared to the expression of control cells transfected with an empty pCAG plasmid + shRNA scramble or with the Sp8 expressing plasmid alone. These interfering experiments reveal a higher efficiency of shRNA 9 (Relative expression of Sp8 mRNA expression in cells transfected with the control Sp8 plasmid: 100±6.494 (n=7) vs cells transfected with the Sp8 plasmid + the shRNA9: 37.03±7.538 (n=4)). (D) 72 h post transfection, transfected HEK cells were fixed and immunostained against Sp8. An optical densitometric analysis confirmed a decreased Sp8 expression following shRNA9 electroporation. Relative expression level of Sp8 in cells transfected with the control Sp8 plasmid: 100±6.499 vs cells transfected with the Sp8 plasmid + the shRNA9: 73.86±6.794 (n=20). shRNA9, which was therefore chosen for all in vivo experiments. Error bars represent the standard error of the mean; **p<0.01; ***p<0.001 determined by unpaired t-test.

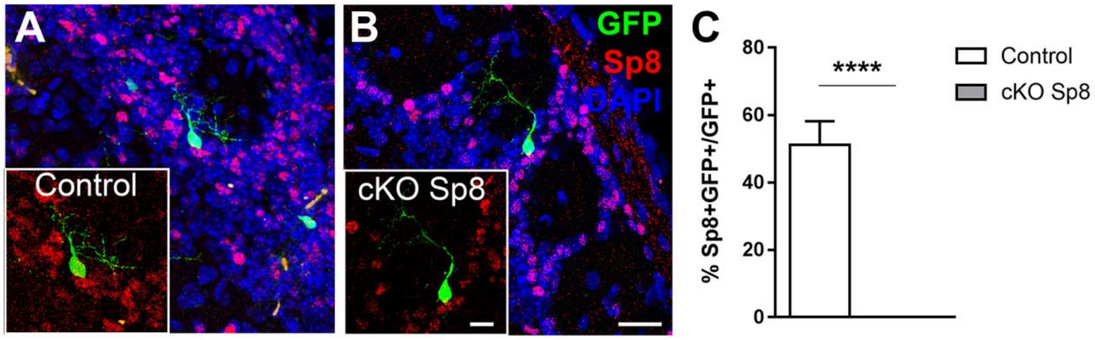

Supplementary figure S4: Efficiency of Permanent Sp8 deletion in NSCs and their progeny.

(A-B) Representative immunodetection of Sp8 in PG GFP+ electroporated cells at 21dpe in control (A) and cKO Sp8 animal (B). Scale bars: 10µm and 20µm. (C) Proportion of electroporated PG neurons expressing Sp8 at 21 dpe following medial EPO in control and cKO animals. Error bars represent the standard error of the mean; ****p≤0.0001 determined unpaired t-test.
